# Plasmid Profile and Pulsed–Field Gel Electrophoresis Analysis of *Salmonella enterica* Isolates from Humans in Turkey

**DOI:** 10.1371/journal.pone.0095976

**Published:** 2014-05-22

**Authors:** Kerem Ozdemir, Sumeyra Acar

**Affiliations:** 1 Yuzuncu Yıl University, Faculty of Science, Department of Biology, Van, Turkey; 2 Public Health Institution of Turkey, Ankara, Turkey; St. Petersburg Pasteur Institute, Russian Federation

## Abstract

This study was conducted for typing *Salmonella enterica* subspecies *enterica* strains in Turkey using pulsed–field gel electrophoresis (PFGE) and plasmid DNA profile analysis. Fourty-two strains were isolated from clinical samples obtained from unrelated patients with acute diarrhea. The samples were collected from state hospitals and public health laboratories located at seven provinces in different regions of Turkey at different times between 2004 and 2010. The strains were determined to belong to 4 different serovars. The *Salmonella enterica* strains belonged to the serovars *Salmonella* Enteritidis (n = 23), *Salmonella* Infantis (n = 14), *Salmonella* Munchen (n = 2), and *Salmonella* Typhi (n = 3). Forty-two *Salmonella enterica* strains were typed with PFGE methods using *Xba*I restriction enzyme and plasmid analysis. At the end of typing, 11 different PFGE band profiles were obtained. Four different PFGE profiles (type 1, 4, 9, and 10) were found among serotype *S.* Enteritidis species, 3 different PFGE profiles (type 3, 5, 6) were found among *S.* Infantis species, 2 different PFGE profiles were found among *S.* Typhi species (type 2 and 11), and 2 different PFGE profiles were found among *S.* Munchen species (type 7, 8). The UPGMA dendrogram was built on the PFGE profiles. In this study, it was determined that 4 strains of 42 *Salmonella enterica* strains possess no plasmid, while the isolates have 1–3 plasmids ranging from 5.0 to 150 kb and making 12 different plasmid profiles (P1–P12). In this study, we have applied the analysis of the PFGE patterns and used bioinformatics methods to identify both inter and intra serotype relationships of 4 frequently encountered serotypes for the first time in Turkey.

## Introduction

*Salmonella enterica* is one of the major causative pathogens of food borne disease outbreaks [1] and also a public health concern all over the world [2]. It has increased dramatically in European countries, and it causes an estimated 17 million annual cases of acute gastroenteritidis or diarrhea according to the World Health Organization [3], [4]. Nontyphoidal *Salmonella* kills 3 million children each year in both developed and developing countries [2]. *Salmonella* includes more than 2,400 different known serovars, and *Salmonella enterica* serovar Enteritidis is one of the most common causes of human gastroenteritidis according to Centers for Disease Control and Prevention. Since 1993, the two most frequent serovars reported are *S.* Enteritidis and *S.* Typhimurium [5]. More than 90% of *Salmonella* strains isolated from humans in Turkey in the 1970's was *S.* Typhimurium [6]. Although *S.* Typhimurium has been featured as the most common serotype until recently, incidence of *S.* Enteritidis is gradually increasing and it has been the serotype isolated most frequently in the last 10 years. However, there are very few researches that investigate the state and regional differences throughout Turkey [7].

*Salmonella* species are often found in red meats, milk, and dairy products [8]. Particularly in recent years, *Salmonella* cases have increased in Turkey due to the increase in national and international dairy product trade in many regions of the world and changes in food production and consumption habits. For this reason, it is necessary to search the features of *Salmonella* roots found in Turkey in order to identify which strains are suitable for typing. Even if phenotypic methods are useful in epidemiological researches, the application of many of them in research remains controversial [9]. Although molecular methods are adequately distinctive in classifying *Salmonella* serotypes into subspecies in epidemiological studies, very few molecular studies have been conducted in Turkey. Among the molecular-based techniques used recently, plasmid profile analysis [10], [11], random amplified polymorphic DNA analysis (RAPD) [11], repetitive extragenic palindromic sequences analysis by PCR (rep-PCR) [12], and pulsed–field gel electrophoresis (PFGE) [10], [13], [14] are commonly used. Chromosomal fingerprinting by means of pulsed–field gel electrophoresis is considered as a gold standard method for subtyping and the most reliable among molecular techniques [15]. PFGE with endonuclease *Xba*I has been widely recognized as a sensitive means for epidemiological analysis of *Salmonella* serovars [16].

The objective of this study was to evaluate the characteristics of *Salmonella* serovars isolated from humans in Turkey by plasmid profile analysis and PFGE. To the best of our knowledge, this is the first study on different *Salmonella* isolates from Turkey using plasmid profile analysis and PFGE methods.

## Materials and Methods

### Bacterial strains

In this study, strains isolated from 42 clinical non-related samples with acute diarrhea were randomly collected from state hospitals and public health laboratories located at seven provinces in different regions of Turkey (Izmir, Bursa, Ankara, Istanbul, Van, Erzurum, Igdır). These strains were isolated from human feces. Samples were collected between 2004 and 2010, and they were chosen as isolates from sporadic cases (just two strains, st 36 and st 37, were from the outbreak that occurred 2008 in Van). Different geographical locations are listed in Table 1 by date. Isolates were taken as *Salmonella* spp., their biochemical and serological typing was done in accordance with the standard laboratory methods.

**Table 1:** Distribution of *Salmonella enterica* serovars.

*Salmonella* serovars	Year	Origin	Strain no	Total
*S.* Enteritidis	2004	Ankara	st26	1
	2006	Van	st16	1
	2008	İstanbul	st24	1
	2009	Erzurum	st 11,12,13	3
	2009	Ankara	st 14,18,23	3
	2009	Van	st17	1
	2009	İzmir	st25	1
	2010	Ankara	st 5,6,15,21	4
	2010	İstanbul	st 7,22	2
	2010	Bursa	st 8,9,19,27	4
	2010	İzmir	st 10,20	2
*S.* Infantis	2008	Ankara	st 40	1
	2008	İstanbul	st 1	1
	2008	Van	st 38	1
	2006	Van	st 28	1
	2004	İstanbul	st 42	1
	2005	Erzurum	st 39	1
	2005	İzmir	st31	1
	2009	Ankara	st 2,41	2
	2009	İstanbul	st 3	1
	2010	Ankara	st 4	1
	2010	Bursa	st 29,30,32	3
*S.* Typhi	2008	Van	35,36,37	3
*S.* Munchen	2007	Igdır	33	1
	2010	Bursa	34	1

### Serotyping

Stool samples suspected to contain *Salmonella* were obtained at different times and subjected to incubation for 18–24 h at 37°C. Identification was performed using standard biochemical methods by selecting suspicious colonies, and lam agglutination method was applied by using polyvalent-monovalent *Salmonella* somatic 0 and flagella H antisera, and was serotyped according to Kauffmann-White schema, and finally stocked and stored at −80°C.

### Antimicrobial susceptibility testing

Antimicrobial susceptibilities for *Salmonella* spp. isolates were performed by the standard disk diffusion method in Mueller-Hinton agar, as per the methods of the Clinical and Laboratory Standards Institute. All the strains were tested for resistance to the following 21 antibiotics (Oxoid, England)): Ampicillin (AMP) (10 µg), cephalotin (KF) (30 µg), gentamicin (CN) (10 µg), amoxicillin-clavulanicacid (AMC) (25 µg), cephuroxim sodium (CXM) (30 µg), cephoperazone (CFP) (30 µg), cephotaxim (CTX) (30 µg), ceftizoxime (ZOX) (30 µg), ceftriaxone (CRO) (30 µg), ceftazidim (CAZ) (30 µg), sulfamethoxazole/trimethoprim (SXT) (25 µg), chloramphenicol (C) (30 µg), tetracycline (TE) (10 µg), canamycin (K) (30 µg), nalidixic acid (NA) (30 µg), ciprofloxacin (CIP) (5 µg), sulfonamides (S3) (30 µg), streptomycin (S10) (10 µg), trimethoprim (W) (25 µg), cefpodoxim (CPD) (10 µg), and amikacin (AK) (30 µg). *E. coli* (ATCC 25922) was used as a quality control strain.

### Plasmid profile analysis

Plasmids were obtained using the method of Kado and Liu with modifications [17]. Seven percent agarose gel (Sigma Aldrich, USA) containing 0.5 µg ethidium bromide was used for the analysis with 0.5× Tris-Boric acid–EDTA buffer at 110 V for 3 h. Plasmid lengths and plasmid sizes were determined with known *E. coli* R39 (147 kbp, 63 kbp, 36 kbp) and supercoiled DNA ladder (Invitrogen, Carlsbad, CA).

### Pulsed–field gel electrophoresis

Analysis of isolates using PFGE method was done in accordance with CDC (Centers for Disease Control and Prevention) Pulse Net protocol (www.cdc.gov/pulse.net). Agarose plugs were prepared by cutting genomic DNA with 50 U *Xba*I (Fermentas Life Sciences, St. Leon-Rot,Germany) restriction enzyme. Electrophoresis was performed with 1% SeaKem Gold agarose gel in 0.5× Tris-Boric Acid-EDTA buffer using the CHEF DRII system (Bio-Rad, Hercules, USA) for 19.4 h with switch times of 2.2–63.8 at 6 V/cm, 14°C. The gels were stained with ethidium bromide (2 mg/mL, Sigma) for 25 min and washed thrice with distilled water for 15 min and visualized using UV transilluminator. The restriction patterns were compared by using the Bionumerics version 6.01 software with the Dice coefficient with 1.5% band tolerance and 1% optimization and the unweighted pair group method with arithmetic averages (UPGMA). Clinical isolates were grouped based on the similarity with a coefficient higher than 85% to show clonal relationships.

## Results

### *Salmonella* serotypes

In this study, 42 *Salmonella enterica* isolates that were different from each other were identified. After serotyping, 4 different serovars were determined: *S.* Enteritidis (23), *S.* Infantis (14), *S.* Typhi (3), and *S.* Munchen (2). The dominant serovar was *S.* Enteritidis, which is the most frequently encountered serotype in human infections in Turkey as of 1990, while others were accepted as minor serovar.

### Antimicrobial susceptibility

The results were evaluated according to the suggestions of Clinical and Laboratory Standards Institute (CLSI). The pattern of resistance of the *Salmonella* analyzed in this study is shown in Table 2. The antimicrobial resistance profiles were different among serovars of the 42 *Salmonella* clinical isolates, and 30.3% (42/14) were resistant to one or more antimicrobials. Sixteen *S.* Enteritidis isolates were sensitive to all antibiotics tested. Multi-drug resistance was higher in *S.* Infantis strains than in the other serovars. The highest rates of resistance were observed for ampicillin and nalidixic acid. Antimicrobial susceptibility results are given in Table 2.

**Table 2:** Antibiotic resistance profiles.

*Salmonella* Serovars	Resistance pattern	No. of strains	Total No
**no. of strains**			
Enteritidis (23)	NA	1	7
	NA+AMP	1	
	AMP	5	
Infantis (14)	TE+AMP	2	6
	NA+SXT+AMP	1	
	TE+AMP+NA	3	
Typhi	SXT+AMP+C	1	1

NA, nalidixic acid; AMP, ampicillin; TE, tetracycline; SXT; sulfamethoxazole-trimethoprim.

### Plasmid DNA profile analysis

It was determined that 4 of the 42 *Salmonella enterica* strains examined (9.3%) had plasmid, and 1 of them belonged to the *S.* Enteritidis serotype, one belonged to *S.* Munchen, and 2 belonged to the *S.* Typhi serotype. Isolates carrying plasmid (90.6) had 1–4 plasmids whose size ranged between 5.0 and 150 kb. Twelve plasmid DNA profile was found in strains with plasmid (type1–type 12). Serotype *S.* Enteritidis isolates had 6 different plasmid DNA profiles, and all of them had 57 kb of plasmid (type 2, type 3, type 4, type 5, type 6, and type 7). Among the *Salmonella* isolates, 30.4% were found in type 2 (57 kb; 3.7 kb); 21.7% in type 3 (57 kb; 3.7 kb; 3.4 kb), 34.7% in type 4 (57 kb), 4.3% in type 7 (57 kb; 30 kb; 3.7 kb), and 4.3% in type 6 (57 kb; 30 kb) plasmid DNA profile type. Moreover, 71.4% of *S. Infantis* isolates were found in type 1 (150 kb), 14.2% type 8 (150 kb; 60 kb), 7.14% type 9 (150 kb; 5.5 kb), and 7.14% type 12 (60 kb) plasmid DNA profile. While one of the *S.* Munchen (67 kb) isolates showed type 10 profile, the other had no plasmid (type 5). While one of *S.* Typhi isolates showed type11 plasmid DNA profile (148 kb), the other 2 isolates did not have a plasmid.

### PFGE analysis

**Figure 1:**
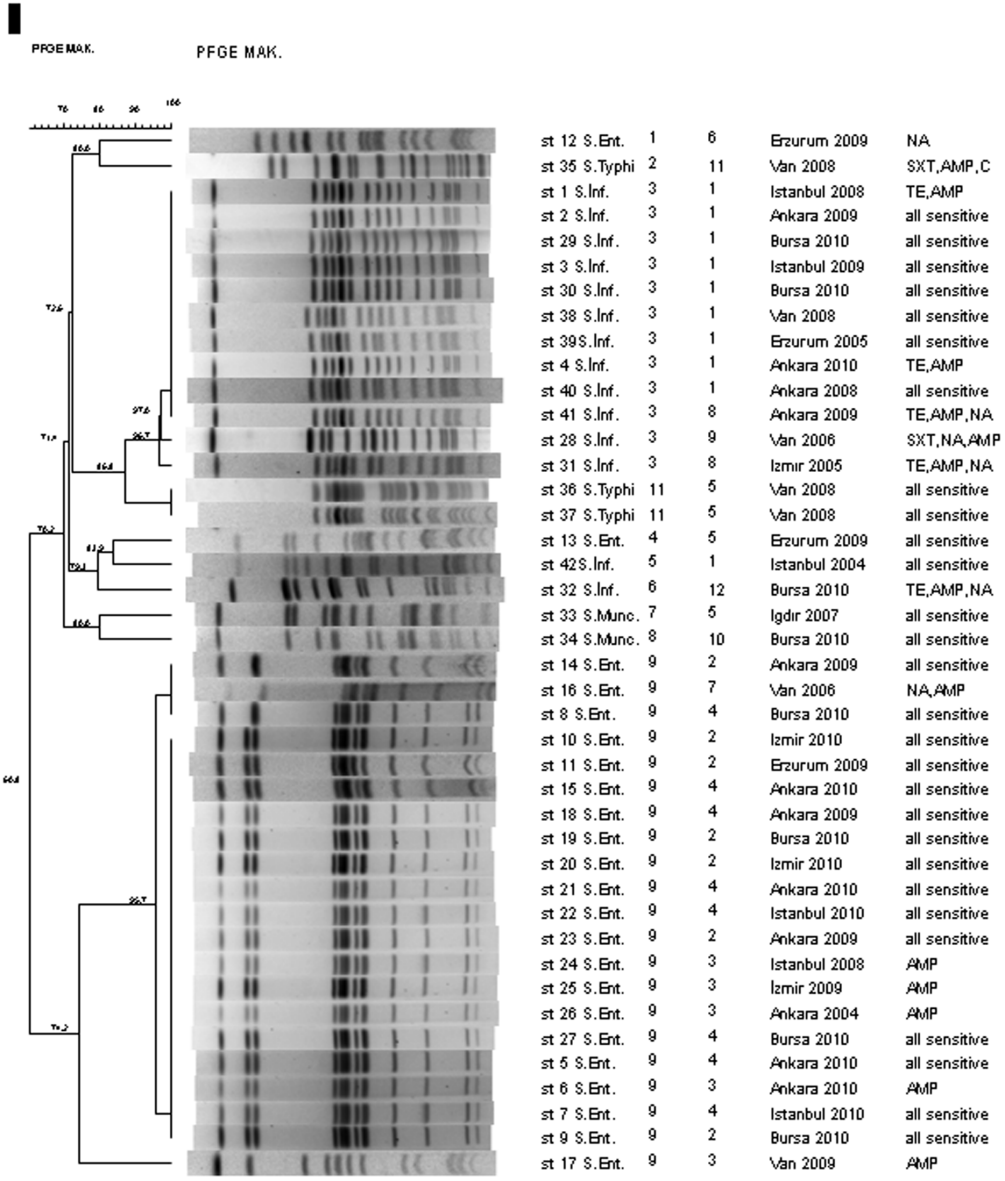
Dendogram of *S. enterica* serovar isolates showing percent similarity has been calculated by the Dice similarity of PFGE (*Xba*I) restriction endonuclease digestion, constructed using UPGMA algorithm (Bionumerics version 6.01 software) by using 1.5% band tolerance and 1% optimization settings.

**Figure 2:**
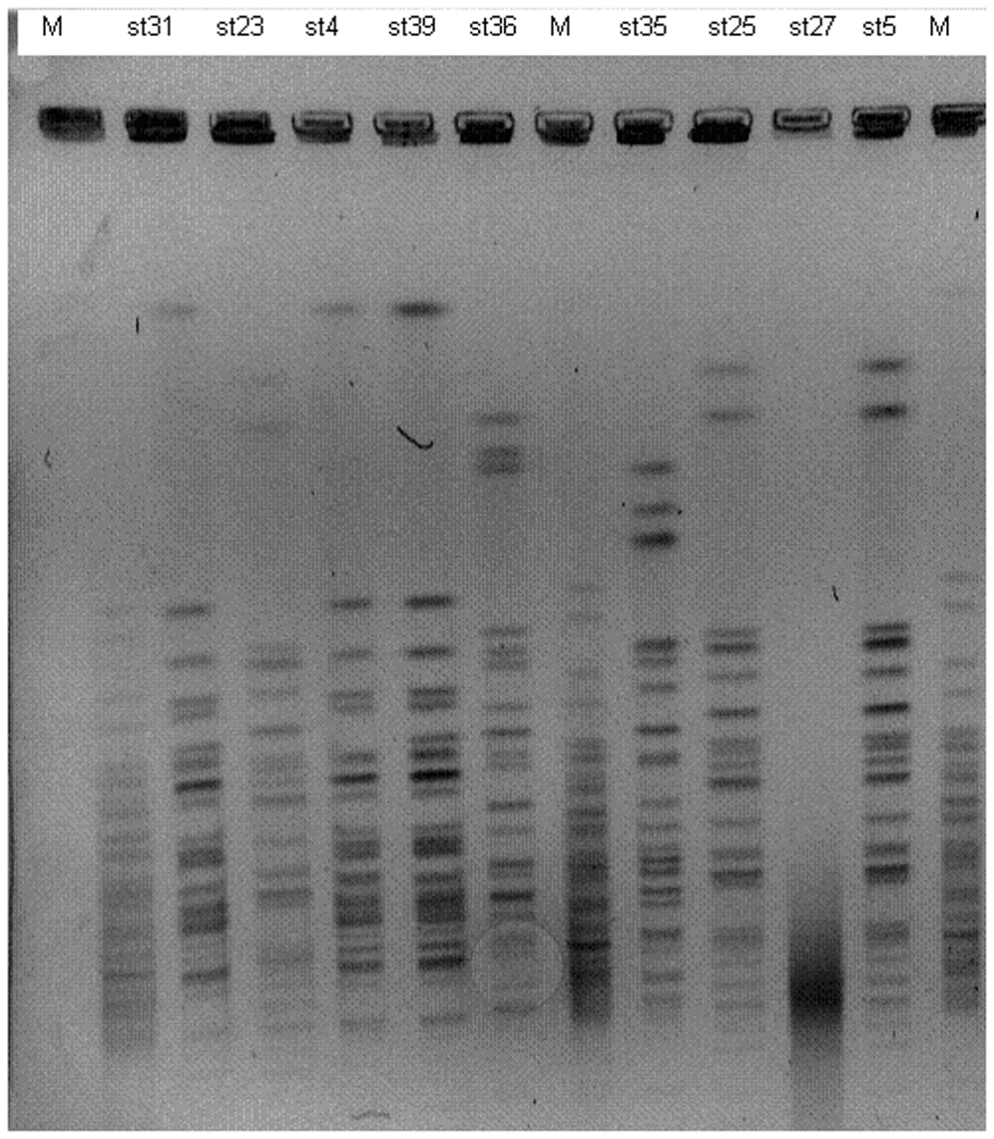
Examples of representative PFGE fingerprinting patterns of *S. enteritidis*, *S. typhi*, and *S. infantis* strains after restriction with SpeI enzymes.st31, st23, st4, st39, st35, st25, st27; M: PFGE marker (Supercoiled DNA, USA).

After *Salmonella enterica* strains were examined by PFGE method and cut using *Xba*I macrorestriction enzyme, bands ranging between 8 and 13 were obtained. Dendogram of PFGE models created using Dice similarity coefficient and UPGMA method is shown in Figure 1. *S.* Enteritidis strain cut with *Xba*I enzyme created 2 different PFGE models containing bands ranging between 11 and 16 (type 1, 9, 4, 10). Among the PFGE types, similarity was noticed below 85% and above 95% in strains within the same type. Three different PFGE types containing band ranging between 15 and 17 were found among *S.* Infantis strains cut with *Xba*I enzyme (type 3, 5, 6). Types show similarity below 85%. While strains belonging to *S.* Munchen serotype had 2 different PFGE type (type 7 and type 8), they showed 80% similarity among them. Strains belonging to *S.* Typhi serotype showed band profile ranging between 15 and 18 and they had 2 different PFGE types (2, 11). Eighty-six percent similarity and 7 band differences were seen between *S.* Infantis strain (st 31) and *S.* Typhi (st 35, st 36) strains and they are closely related. Therefore, dominant *S.* Enteritidis profile (type 9), *S.* Infantis profile (type 3), and closely related st 31 *S.* Infantis and st 35, 36 *S.* Typhi strains were cut with second macrorestriction enzyme (*Spe*I) for confirmation (Figure 2). As a result, Type 1 *S.* Enteritidis and *S.* Infantis strains were similar, but st 31 *S.* Infantis and st 35, 36 were different. *S.* Typhi isolates were undistinguishable with 9 bands difference, and 73% similarity rate.

Plasmid profiles and PFGE patterns are given in Table 3 together with the origins of strains and the year when the strains were isolated. One PFGE and plasmid type specific to each serotype was found, and thus two molecular methods generated compatible results.

**Table 3:** Distribution of plasmid types and pulsed–field gel electrophoresis types together among *Salmonella* serotypes.

*Salmonella* serovars	PFGE type	Plasmid type	Number of strains	Origin
*S.* Enteritidis	type 9	type 2	7	Ankara, Bursa, İstanbul
	type 9	type 3	6	Ankara, İzmir
	type 9	type 4	6	Ankara, Bursa, Van, Erzurum
	type 1	type 5	1	Erzurum
	type 4	type 6	1	Erzurum
	type 10	type 4	1	Van
	type 9	type 7	1	İstanbul
*S.* Infantis	type 3	type 1	10	İstanbul, Ankara, Van, İzmir, Erzurum
	type 5	type 12	1	İstanbul
	type 3	type 8	2	Bursa
	type 6	type 9	1	Bursa
*S.* Typhi	type 2	type 11	1	Van
	type 11	type 5	2	Van
*S.* Munchen	type 7	type 5	1	Igdır
	type 8	type 10	1	Bursa

## Discussion

Non-typhoidal *Salmonella* species are the primary contaminants among food-borne pathogens [18], [19]. It has been reported that *Salmonella* serotype seen most frequently in Turkey and around the world is *S.* Enteritidis (www.cdc.gov/mmwr/2005) [20], and the data generated in our study supports this fact. Abbassi-Ghozzi et al. (2006) examined 32 clinical *Salmonella* and found that 62.5% was *S.* Enteritidis [5]. Similarly, Turkey National Reference laboratory stated that 67.41% of *Salmonella* isolates seen in Turkey belongs to *S.* Enteritidis serotype, 4.41% belongs to *S.* Infantis, 2.5% belongs to *S.* Typhi, and 0.7% belongs to *S.* Munchen serotype, as stated in 2007–2010 data of the national enteric pathogen laboratory [21]. According to the data provided by Turkey national reference laboratory, it was reported that 15.8% of *Salmonella* strains isolated in 2010 belonged to clinical isolates, and 79.4% of these human isolates belonged to *S.* Enteritidis serotype.

Many phenotypic and genotypic methods have been developed and are continued to be developed for typing of *Salmonella* species [22]. Various studies were conducted for serotyping and subtyping of *Salmonella* species and typing powers of the methods were compared. Plasmid DNA profile analysis is a method, which has been used for a number of years, for separation of serovars belonging to *Salmonella* species and subtypes within the serovar [23]. This method demonstrated better separation mainly in serotype typing [5]. *Salmonella* spp. are able to transfer plasmids they carry to other bacteria or they are able to gain plasmid over time and serve as an antibiotic-resistant determinant [24]. In our study, all of the 14 resistant *Salmonella* spp. isolates were seen to have plasmid. Since sensitive isolates could contain plasmids, there is a possibility that these plasmids could carry other antibiotic-resistance genes not tested in this study or be associated with virulence, conjugation, metal resistance, or other significant bacterial traits that have been associated with megaplasmids (5). Thirty-eight of the 42 *Salmonella* isolates carried at least one plasmid. Nevertheless, analysis of plasmid DNA profile alone may be misleading in molecular typing due to similar features. However, validation using another molecular method enhances the reliability of the result. In this work, plasmid DNA profile analysis and PFGE method were used together. PFGE was adopted for national *Salmonella* surveillance and outbreak research in the 1990s, and has been successfully used in typing *Salmonella* from human patients, animal sources, and foods because of its remarkable discriminatory power and high reproducibility [25], [26]. In this study, PFGE provided a better understanding of the genetic relationship, diversity, and epidemiology of human *Salmonella* spp. There are many studies in which PFGE method is employed with different molecular methods. For example, Soyer et al. (2010) employed PFGE and MLST methods together, and they showed that PFGE method has better discrimination power in *Salmonella*. In other works, RAPD method and ribotyping were tried together with PFGE method and it was concluded that PFGE had the best discriminatory power among these methods [27], [28], [29]. However, another study stated that some *Salmonella* strains cannot be typed with PFGE method [5]. In this case, next-generation sequencing (NGS) can be used; it is increasingly being used as a molecular epidemiologic tool for discerning ancestry and to trace back most complicated bacterial pathogens that are difficult to resolve. NGS data acquisition and analysis provides highly reproducible results that are stable and predictable for molecular epidemiological applications [30].

*S.* Enteritidis is the most prominent serotype in *Salmonella* species that is seen most frequently in Turkey and worldwide, and is studied extensively. In a study carried out in Caribbean countries, it was seen that the dominant subtype in PFGE profile, obtained by cutting DNA of isolates belonging to *S.* Enteritidis serotype with *Xba*I restriction enzyme, was similar to the dominant PFGE pattern obtained in this study using the same enzyme [31].

The dominant PFGE type seen in *S.* Enteritidis isolates isolated from clinical and environmental samples in various countries of the world (Canada, Sweden, Cuba etc.) was similar to the dominant subtype in this work [32]. Similar results were found in a study carried out by Ammari et al. in Morocco and also in a multi-centered study conducted in Turkey [13], [16]. Nevertheless, the same pattern was not observed for other serotypes found in our work. In this study, PFGE profiles were created by cutting DNA of *S.* Infantis with *Xba*I restriction enzyme [14]. In a similar study conducted in Brazil, different PFGE patterns were found, and dominant subtypes were different from each other [33]. PFGE profiles found in this work were found in previous studies. However, dominant subtypes were not observed. Merino et al. complemented PFGE typing study with plasmid DNA profile as the second molecular method. In this study, they stated that plasmids in the size of 150 kb and 54 kb were seen frequently. This supports the results of our plasmid DNA profile analysis. The characteristics of serotype *S.* Infantis species is similar to that of *S.* Typhi. In a study conducted by Wu et al. in China, no dominant subtype in PFGE profile was obtained after cutting DNA of *S.* Typhi with *Xba*I restriction enzyme, although it showed diversity [34]. In another study conducted in Canada, a dominant subtype was not found. These data are consistent with our findings, as common PFGE types were found, and a dominant profile specific to a serotype was not seen [35].

Molecular epidemiology-based techniques analyzing chromosomal DNA or plasmids have been found to be useful for typing [36]. In this study, we tried to determine the relation between *Salmonella* serotypes isolated from different provinces in Turkey during 2004–2010. We analyzed the PFGE patterns and used bio-informatics methods to identify both inter and intra serotypes relationships of 4 frequently occurring serotypes together for the first time in Turkey. This study aimed to provide insight on the relation between different *Salmonella* serotypes. Considering that *S.* Typhi is the 5^th^ frequently seen serotype and *Salmonella* infantis is the 4^th^ common serotype in Turkey, more detailed studies are required on the epidemiology of *Salmonella* serotypes posing danger in Turkey and worldwide, as this is an important pathogen.
